# Differential involvement of trigeminal transition zone and laminated subnucleus caudalis in orofacial deep and cutaneous hyperalgesia: the effects of interleukin-10 and glial inhibitors

**DOI:** 10.1186/1744-8069-5-75

**Published:** 2009-12-21

**Authors:** Kohei Shimizu, Wei Guo, Hu Wang, Shiping Zou, Stacey C LaGraize, Koichi Iwata, Feng Wei, Ronald Dubner, Ke Ren

**Affiliations:** 1 Department of Neural and Pain Sciences, Dental School, University of Maryland, Baltimore, MD 21201, USA; 2 Program in Neuroscience, University of Maryland, Baltimore, MD 21201, USA; 3 Department of Physiology, Nihon University School of Dentistry, 1-8-13 Kandasurugadai, Chiyoda-ku, Tokyo 101-8310, Japan

## Abstract

**Background:**

In addition to caudal subnucleus caudalis (Vc) of the spinal trigeminal complex, recent studies indicate that the subnuclei interpolaris/caudalis (Vi/Vc) transition zone plays a unique role in processing deep orofacial nociceptive input. Studies also suggest that glia and inflammatory cytokines contribute to the development of persistent pain. By systematically comparing the effects of microinjection of the antiinflammatory cytokine interleukin (IL)-10 and two glial inhibitors, fluorocitrate and minocycline, we tested the hypothesis that there was a differential involvement of Vi/Vc and caudal Vc structures in deep and cutaneous orofacial pain.

**Results:**

Deep or cutaneous inflammatory hyperalgesia, assessed with von Frey filaments, was induced in rats by injecting complete Freund's adjuvant (CFA) into the masseter muscle or skin overlying the masseter, respectively. A unilateral injection of CFA into the masseter or skin induced ipsilateral hyperalgesia that started at 30 min, peaked at 1 d and lasted for 1-2 weeks. Secondary hyperalgesia on the contralateral site also developed in masseter-, but not skin-inflamed rats. Focal microinjection of IL-10 (0.006-1 ng), fluorocitrate (1 μg), and minocycline (0.1-1 μg) into the ventral Vi/Vc significantly attenuated masseter hyperalgesia bilaterally but without an effect on hyperalgesia after cutaneous inflammation. Injection of the same doses of these agents into the caudal Vc attenuated ipsilateral hyperalgesia after masseter and skin inflammation, but had no effect on contralateral hyperalgesia after masseter inflammation. Injection of CFA into the masseter produced significant increases in N-methyl-D-aspartate (NMDA) receptor NR1 serine 896 phosphorylation and glial fibrillary acidic protein (GFAP) levels, a marker of reactive astrocytes, in Vi/Vc and caudal Vc. In contrast, cutaneous inflammation only produced similar increases in the Vc.

**Conclusion:**

These results support the hypothesis that the Vi/Vc transition zone is involved in deep orofacial injury and suggest that glial inhibition and interruption of the cytokine cascade after inflammation may provide pain relief.

## Background

Sensory information from the cranial orofacial region is first relayed in the spinal trigeminal nucleus complex, which is further divided rostrocaudally into the subnuclei oralis, interpolaris (Vi) and caudalis (Vc) [1]. It is widely accepted that nociceptive input from the cranial orofacial region is initially processed in the Vc [2], which exhibits lamination and considerable similarity with spinal dorsal horn and thus is termed the medullary dorsal horn [3]. Advances in our understanding of trigeminal pain processing have occurred in recent years and attention has been given to other components of trigeminal nociceptive pathways beyond the medullary dorsal horn [4-6]. Particularly, studies have pointed out increased excitability and sensitization of another region of the spinal trigeminal complex, the Vi/Vc transition zone. Around the obex level, the ventral portion of the laminated Vc is replaced by the caudal Vi that converges with the rostral Vc with imperfectly laminated structures, allowing the appearance of the Vc (mainly dorsal) and Vi (mainly ventral) at the same coronal plane and thus termed the trigeminal Vi/Vc transition zone [see [7]]. Most interestingly, a peculiar bilateral neuronal activation in the ventral portion of the Vi/Vc transition zone, together with unilateral activation in the caudal Vc, has been observed following orofacial injury and noxious stimulation [8-14]. Further studies suggest that the Vi/Vc transition zone is involved in processing deep orofacial input. Utilizing Fos protein expression as a marker of neuronal activation, it has been shown that deep tissue masseter inflammation evokes activity in the Vi/Vc and caudal Vc regions, whereas after cutaneous injury, activity is almost entirely limited to the caudal Vc [11]. While both masseter and cutaneous inputs project to the caudal Vc, masseter, but not cutaneous, afferents provide an additional input to the Vi/Vc [7].

Recent studies suggest that glia and inflammatory cytokines contribute to the development of persistent pain [15-20]. In the spinal dorsal horn, it has been found that numerous glial profiles, particularly astrocytic profiles, are in apposition with descending serotonergic and noradrenergic varicosities [21]. Peripheral tissue or nerve injury induces central nervous system (CNS) glial hyperactivity, mainly involving astrocytes and microglia [22,23]. Earlier evidence indicates that spinal astrocytes are activated after nerve injury [24,25]. Activation of microglia has been shown to play a critical role in neuropathic pain [23,26-29]. Disrupting glial activation blocks exaggerated pain responses and activation of glia is sufficient to induce hyperalgesia [30]. Intrathecally administered IL-1β, a prototypical proinflammatory cytokine, produces enhanced spinal dorsal horn nociceptive neuronal responses and behavioral hyperalgesia [31-33]. In contrast, anti-inflammatory cytokines, such as interleukin (IL)-10, block the induction of proinflammatory cytokines and attenuate hyperalgesia [34-36]. Laughlin et al. [37] demonstrated that IL-10 attenuated intrathecal dynorphin-induced allodynia. Our recent results have demonstrated that in association with astroglial activation, IL-1β is induced in the Vi/Vc transition zone after masseter inflammation and that the development of orofacial hyperalgesia involves signal interactions between the IL-1 receptor and the N-methyl-D-aspartate (NMDA) receptor [17].

By systematically comparing the effects of focal microinjection of the antiinflammatory cytokine IL-10 and two glial inhibitors, fluorocitrate and minocycline, the present study tested the hypothesis that there was differential involvement of Vi/Vc and caudal Vc structures in deep and cutaneous orofacial pain. The results show that injection of IL-10 and glial inhibitors into the Vi/Vc attenuated masseter but not cutaneous hyperalgesia, while injection of these agents into the Vc reduced both masseter and cutaneous hyperalgesia associated with complete Freund's adjuvant (CFA)-induced inflammation.

## Methods

### Animals

Male Sprague-Dawley rats were used (280-330 g, Harlan, Indianapolis). The rats received CFA (0.05 ml, 1:1 oil/saline suspension, Sigma) into the unilateral masseter muscle or the skin overlying the masseter muscle under brief halothane anesthesia to induce inflammation and hyperalgesia. Saline-injected and naive rats were used as controls. The selective inflammation of the muscle and cutaneous tissues was verified with Evans' blue extravasation as described previously [7]. After injection of CFA into the masseter muscle, the skin overlying the inflamed masseter did not show an increase in the dye level compared to the naive rats, whereas the Evans' blue concentration was increased in the masseter. Injection of CFA into the skin overlying the masseter muscle led to an increase in the Evans blue dye level in the skin but not in the deep masseter muscle. The CFA-injected rats show normal behavior and levels of activity and the effect of hyperalgesia on normal behavior of the animal was minimal [10,11,38,39]. All experiments were carried out in accordance with the National Institute of Health Guide for the Care and Use of Laboratory Animals (NIH Publications No. 80-23) and approved by the University of Maryland Dental School Institutional Animal Care and Use Committee. All efforts were made to minimize the number of animals used and their suffering.

### Behavioral testing

**Figure 1:**
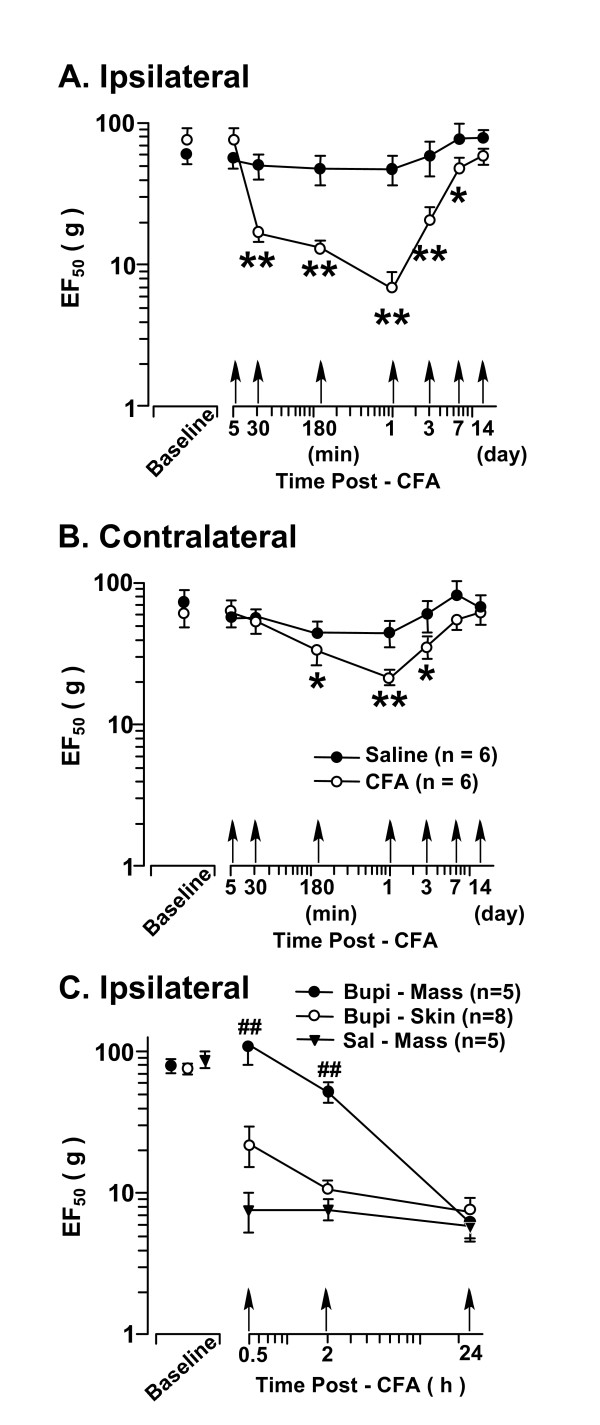
**Mechanical hyperalgesia/allodynia induced by injection of CFA into the right masseter muscle of the rat**. The EF_50_s were derived from the respective stimulus-response frequency function curves and are plotted against time. Note log scales for the ordinate and abscissa. **A**. Ipsilateral site. Note significant decreases in EF_50_s at 30 min-7 d after CFA injection, indicating inflammatory hyperalgesia/allodynia. **B**. Contralateral site. There were significant reductions in EF_50_s at 180 min-3 d after CFA, indicating the development of secondary hyperalgesia after masseter inflammation. **C**. To verify muscle hyperalgesia after injection of CFA into the masseter, local anesthesia was induced in the masseter or the overlying skin ipsilateral to inflammation by bupivacaine (0.75%, 0.1 ml) at 20 min before CFA injection. Compared to saline-treated rats (Sal Mass, n = 5), hyperalgesia was eliminated when the masseter was anesthetized (Bupi Mass, n = 5) and reappeared at 24 h after CFA. Local anesthesia of the skin overlying masseter (Bupi Skin, n = 8) did not produce a significant effect on the development of hyperalgesia. Asterisks in panels A and B denote significant differences from the baseline values (*, p < 0.05; **, p < 0.01). Pound signs in C indicate significant differences from the saline control (Sal Mass) (p < 0.01). (ANOVA with repeated-measures and *post hoc *tests).

All behavioral tests were conducted under blind conditions as described elsewhere [14,40]. A series of calibrated von Frey filaments were applied to the skin above the masseter muscle. An active withdrawal of the head from the probing filament was defined as a response. Each von Frey filament was applied 5 times at intervals of a few sec. The response frequencies [(number of responses/number of stimuli) × 100%] to a range of von Frey filament forces were determined and a stimulus-response (S-R) curve plotted. After a non-linear regression analysis, an EF_50 _value, defined as the von Frey filament force (g) that produces a 50% response frequency, was derived from the S-R curve. We used EF_50 _values as a measure of mechanical sensitivity. A leftward shift of the S-R curve, resulting in a reduction of EF_50_, occurred after inflammation [14,41]. This shift of the curve suggests the presence of mechanical hyperalgesia and allodynia since there was an increase in response to suprathreshold stimuli and a decreased response threshold for nocifensive behavior. Local anesthesia of the skin site overlying the inflamed masseter muscle was performed to confirm the validity of the von Frey method for assessing the mechanical sensitivity of the masseter muscle after inflammation (see results and Fig. 1).

### Drug administration

**Figure 2:**
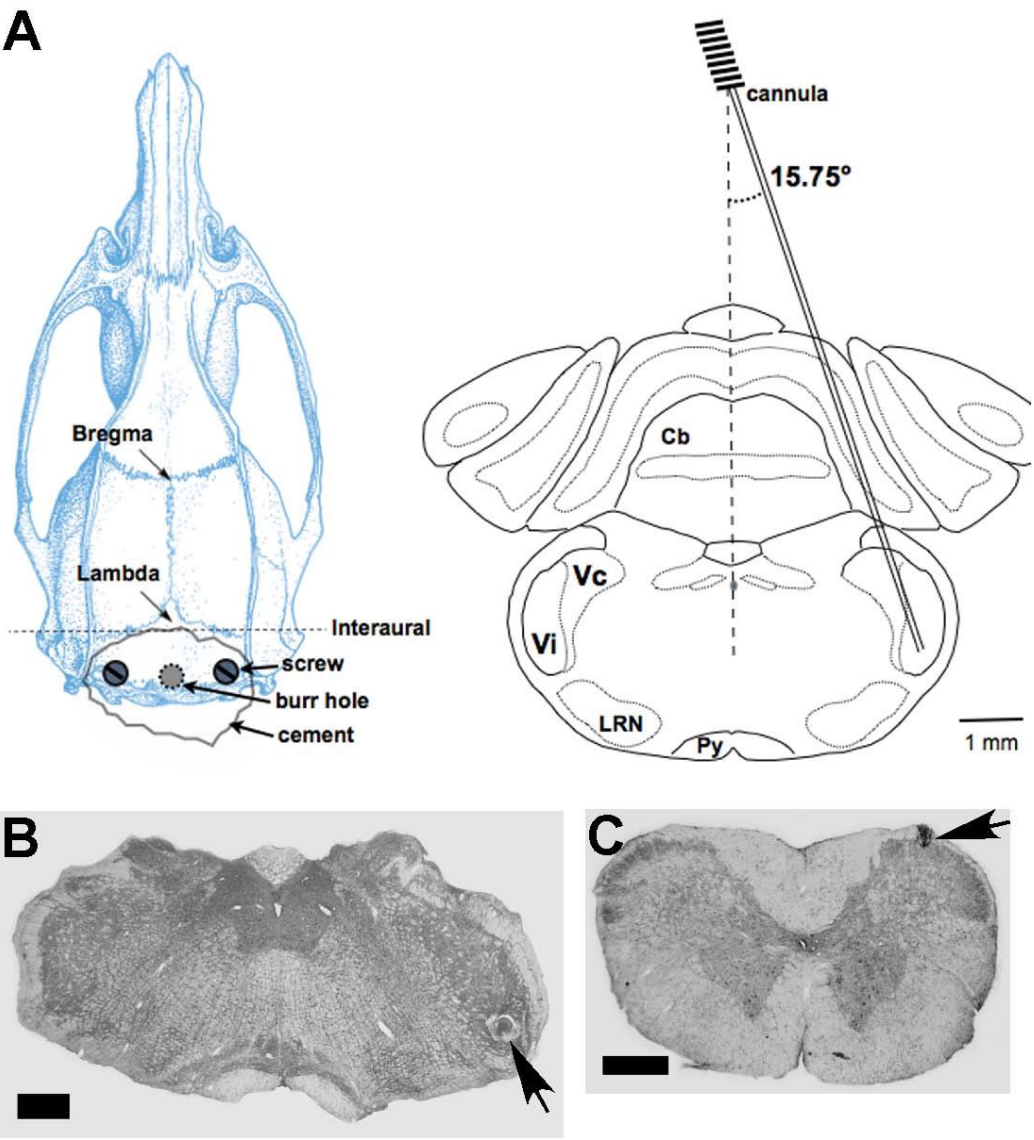
**A. Illustration of implantation of the injection cannula into the Vi/Vc region**. Left: Horizontal view of a rat skull diagram illustrating the site of cannula implantation (burr hole: 4.7-5.2 mm posterior to lambda) [42]. Note that two screws and a patch of cranioplastic cement were used to help secure the cannula. Right: Coronal brain section. The guide cannula was inserted into the brainstem site at a 15.75° angle. The tip of the cannula was located in the ventral Vi/Vc transition zone at a horizontal plane 9.1 mm ventral to the surface of the skull. **B**, **C**. Cresyl violet stained brainstem sections illustrating the site of microinjection in the Vi/Vc transition zone (B) and Vc (C) (arrows). Scale bars in B, C = 0.5 mm.

For microinjection, a guide cannula (C315G, 26 gauge, Plastics One, Roanoke, VA) was implanted under anesthesia with 50 mg/kg pentobarbital sodium (i.p) (Fig. 2A). Animals were securely placed into a stereotaxic device (Kopf Model 900). A burr hole was drilled and the guide cannula was lowered into the ventral Vi/Vc transition zone or superficial layers of the caudal Vc by referring to the rat brain atlas [42]. The guide cannula was then secured with cranioplastic cement. To prevent clogging of the guide cannula, a dummy cannula (C315DC, Plastics One) was inserted and secured in place until the time of injection. The wound was cleaned with an antiseptic solution and closed with 4-0 silk sutures. Animals were allowed to recover for 1 week before further experimentation. Drugs were injected into the ventral Vi/Vc transition zone or superficial Vc ipsilateral to inflammation through a 33-gauge injection cannula (C315I, Plastic One) inserted through the tip of the guide cannula. The injection cannula was connected to a 1-μl Hamilton syringe by polyethylene-10 tubing. All injections (500 nl) were performed by delivering drug or vehicle solutions slowly over a 2-min period. Animals were perfused with 4% paraformaldehyde at the conclusion of the experiment and the sections of brainstem tissues were stained with cresyl violet for histological verification of the sites of injection.

### Brainstem tissue dissection

The rats were anesthetized with halothane and quickly decapitated. A block of caudal brainstem tissues was cut. Two tissue blocks, including a 2-mm segment at the level of the obex and a 2-mm segment at caudal Vc (3-5 mm caudal to the obex), were dissected. The full length of the trigeminal Vi/Vc transition zone is enclosed in the rostral block. The caudal block includes the caudal laminated Vc. The tissue blocks were then turned coronally and the ventral portions of the transition zone and superficial portion of the Vc were harvested by taking punches with a 15-gauge puncture needle.

### Reverse transcription polymerase chain reaction (RT-PCR)

The brain stem tissues were dissected and put immediately into dry ice. Total RNA was extracted after phase separation, precipitation, and ethanol wash. A 260/280 ratio of the final RNA solution was no less than 1.6. The first-strand cDNA was reverse-transcribed in reaction mixture (20 μl) with the SuperScript III First-Strand Synthesis System for RT-PCR (Invitrogen) (Gibco BRL) and oligo-dT primers using 3.0 μg of total RNAs at 42°C for 1 hr. The oligonucleotide primers for PCR reaction were synthesized by The University of Maryland Biopolymer/Genomics Core Facility.

The PCR reaction mixture (50 μl) contained: synthesized cDNA reverse-transcribed using 0.45 μg of total RNAs, 1 × PCR buffer (in mM: 10 Tris-HCl, pH 9.0 at 20°C, 50 KCl, and 1.5 MgCl2), dCTP, dGTP, dATP and dTTP (0.2 mM each), 0.2 mM concentration of each set of the 5' and 3' sequence-specific target primers, recombinant *Taq *DNA Polymerase (Invitrogen) (5 units). PCR was performed after initial denaturation at 94°C for 10 min. The temperature cycle (Robocycler Infinity, Stratagene) was: 94°C/1 min (denaturing), 55°C/1 min (annealing), 72°C/2.5 min (extension). A total of 40 cycles and a final 10 min extension at 72°C were conducted. The glyceraldehyde phosphate dehydrogenase (GAPDH) mRNA RT-PCR was used as an endogenous internal control since its expression was not regulated by inflammation and manipulation within the design of these experiments [43,44]. To minimize the substrate competition [45], the primers for GAPDH cDNA (0.1 mM) were added to the reaction mixture after the first 20 cycles of PCR reaction, and an additional 20 cycles of amplification were performed. The PCR amplified fragments were separated on a 2% ethidium bromide-stained agarose gel. To determine relative expression levels of each target gene, the intensity of each specific PCR product band (UN-SCAN-IT gel 5.3, Silk Scientific Corp.) was normalized to the intensity of the respective GAPDH band and the ratios were used for ANOVA. The positive PCR bands were purified (Agarose Gel DNA Extraction kit, Roche) and sequenced (ABI 373 DNA Sequencer, Perkin Elmer), and the acquired sequences were verified. Mock RT-PCR reaction controls were performed by omitting primers, using templates derived from reverse transcription reactions lacking either reverse transcriptase or total RNAs. No specific PCR product was found in control reactions.

### Western blot

The tissues were homogenized in solubilization buffer (50 mM Tris.HCl, pH8.0; 150 mM NaCl, 1 mM EDTA, 1% NP40, 0.5% deoxycholic acid, 0.1% SDS, 1 mM Na3VO4, 1 U/ml aprotinin, 20 μg/ml leupetin, 20 μg/ml pepstatin A). The homogenate was centrifuged at 20,200 × g for 10 min at 4°C. The supernatant was removed. The protein concentration was determined using a detergent-compatible protein assay with a bovine serum albumin standard. Each sample contains proteins from one animal. The proteins (50 μg) were separated on a 7.5% SDS-PAGE gel and blotted to a nitrocellulose membrane (Amersham Biosciences, Arlington Heights, IL). The blots were blocked with 5% milk in tris-buffered saline (TBS) buffer and then incubated with the respective antibody. The membrane was washed with TBS and incubated with anti-goat or mouse IgG (1:3000, Santa Cruz Biotechnology, Santa Cruz, CA). The immunoreactivity was detected using Enhanced Chemiluminescence (ECL, Amersham). The loading and blotting of the amount of protein was verified by reprobing the membrane with anti-β-actin antiserum (Sigma) and with Coomassie blue staining.

### Drugs and antibodies

The following drugs and antibodies were purchased from commercial sources: bupivacaine (Henry Schein, Melville, NY), IL-10 (PeproTech Inc. Rocky Hill, NJ), fluorocitrate and minocycline (Sigma, St. Louis, MO), and anti-glial fibrillary acidic protein (GFAP) and anti-P-ser896 NR1 antibodies (Millipore-Chemicon, Temecula, CA).

### Data analysis

Data are presented as mean ± S.E.M. Statistical comparisons were made by the use of ANOVA with Fisher's PLSD test for post-hoc analysis. For Western blot and RT-PCR analyses, the ECL-exposed films or gel images were digitized and densitometric quantification of immunoreactive of cDNA bands was carried out using UN-SCAN-IT gel (ver. 5.3, Silk Scientific Inc., Orem, UT). The relative protein or mRNA levels were obtained by comparing the respective specific band to the β-actin or GAPDH control from the same membrane or gels. The deduced ratios were further normalized to that of the naive rats on the same membrane and illustrated as percentage of the naïve controls. ANOVA and the unpaired 2-tailed *t*-test were used to determine significant differences. For animals that were subject to repeated testing, ANOVA with repeated measures was used with time as a within animal effect. P < 0.05 is considered significant for all cases.

## Results

### Mechanical hyperalgesia/allodynia associated with masseter muscle inflammation

Inflammatory hyperalgesia was induced by a unilateral injection of an inflammatory agent, CFA, into the right masseter muscle under brief isoflurane anesthesia. Saline was used as a control. The mechanical responses to von Frey filament probing were assessed and stimulus-response frequency (S-R) curves generated [not shown] [14,41]. After injection of CFA into the masseter muscle, there was a leftward shift of the S-R frequency curve. This shift of the curve suggests the presence of mechanical hyperalgesia and allodynia since there was an increase in response to suprathreshold stimuli and a decreased response threshold to a level that did not in naive animals produce a nocifensive behavior. Consistently, on the side ipsilateral to CFA injection, the derived EF_50_s started to decrease at 30 min (p < 0.01), reached its peak at 1 d (p < 0.01), and returned to the baseline level at 14 d time points (Fig. 1A). An increased mechanical sensitivity also developed on the contralateral side (Fig. 1B), which has been observed previously [14]. The decrease in EF_50_s of the contralateral side started at 180 min (p < 0.05), also reached the maximum reduction at 1 d, and returned to the baseline level at 7 d after inflammation (Fig. 1B). The reduced EF_50 _on the contralateral side suggests the development of secondary hyperalgesia after masseter inflammation.

The mechanical sensitivity of the masseter muscle was assessed through probing the overlying skin. Deep tissue primary afferents are activated by this mechanical pressure. A reduction of EF_50 _at the masseter testing site in CFA-injected rats reflects tenderness and increased sensitivity of deep tissue. Since cutaneous afferents may also contribute to the pain-pressure threshold [46], this method does not distinguish between primary muscle hyperalgesia and referred pain to the overlying cutaneous site. To verify muscle hyperalgesia after injection of CFA into the masseter, we induced local anesthesia in the masseter or the overlying skin ipsilateral to inflammation by infiltrating bupivacaine (0.75%, 0.1 ml) 20 min before injection of CFA into the masseter. Ipsilateral to CFA injection, the hyperalgesia was eliminated when the masseter was anesthetized as compared to saline-treated rats (Fig. 1C). In contrast, the inflammation-induced hyperalgesia was only slightly reduced when the overlying skin testing site was anesthetized (Fig. 1C). Thus, masseter inflammation-induced hyperalgesia alone was detectable when the overlying skin site was anesthetized, validating the method for assessing the sensitivity of the masseter.

### The effects of microinjection of IL-10, fluorocitrate and minocycline on orofacial inflammatory hyperalgesia

To administer drugs focally into the Vi/Vc transition zone and Vc, guide cannulas were implanted. One week after the cannulation, CFA was injected into the masseter or the overlying skin after taking baseline responses. At 1 d after CFA, when hyperalgesia/allodynia had peaked, drugs or vehicle were injected into the ventral transition zone or superficial caudal Vc ipsilateral to inflammation in a volume of 500 nl and the mechanical sensitivity reassessed. The sections of brain stem tissues were made and stained with cresyl violet for histological verification of the sites of injection. We confirmed that the drugs were successfully injected into the target sites. Examples of histological sections illustrating the injection sites in the ventral Vi/Vc and Vc are shown in Fig. 2.

#### The effect of IL-10

**Figure 3:**
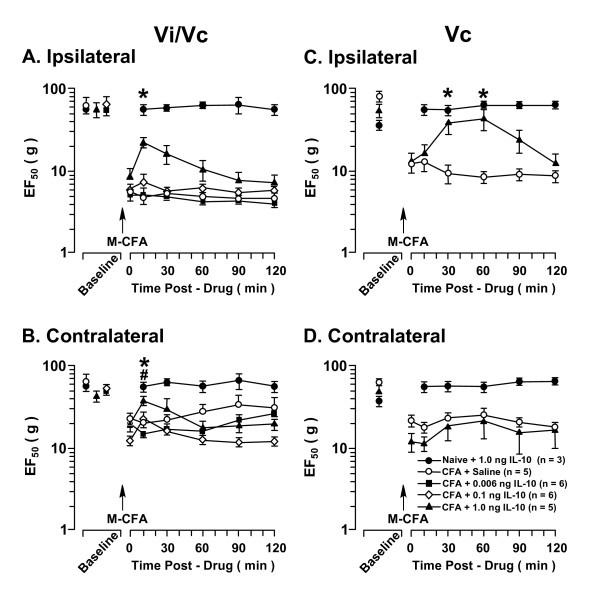
**The effect of IL-10 on orofacial hyperalgesia associated with masseter inflammation**. CFA was injected into the right masseter muscle (M-CFA). At 24 h after CFA, there were significant decreases in the EF_50 _values at both ipsilateral and contralateral sites, confirming the development of hyperalgesia. IL-10 was injected into the Vi/Vc (A, B) or caudal Vc (C, D) in a volume of 500 nl after establishing behavioral hyperalgesia at 24 h after CFA (time 0). The attenuation of hyperalgesia was observed as a significant increase in EF_50_s after injection of IL-10. Note that injection of IL-10 into the Vi/Vc transition zone produced bilateral attenuation of hyperalgesia (A, B), injection of IL-10 into the caudal Vc only attenuated hyperalgesia ipsilaterally (C), and injection of IL-10 into either Vi/Vc or Vc did not produce an effect in non-inflamed (Naive) rats. Asterisks denote significant differences between the post-CFA (time 0) and after 1.0 ng IL-10 microinjection (p < 0.05). Pound sign denotes significant difference between the post-CFA (time 0) and after 0.1 ng IL-10 microinjection (p < 0.05). (ANOVA with repeated-measures and *post hoc *tests).

At 1 d after injection of CFA into the masseter, EF_50_s were significantly reduced bilaterally (p < 0.01) (Fig. 3A, B), confirming the development of hyperalgesia/allodynia (Fig. 1A, B). An antiinflammatory cytokine, IL-10, was injected into the ventral Vi/Vc transition zone at the doses of 0.006 ng (n = 6), 0.1 ng (n = 6) and 1.0 ng (n = 5). The 1.0 ng dose significantly attenuated hyperalgesia/allodynia, as shown by a significant increase in EF_50_s (p < 0.05) (Fig. 3A, B). The effect of the 1.0 ng IL-10 lasted about 30-60 min. The 0.1 ng dose only produced a significant increase in EF50 on the contralateral side (p < 0.05) (Fig. 3B), although there was a trend to produce an increase in EF_50 _on the ipsilateral side (Fig. 3A). The 0.006 ng dose and drug vehicle saline did not affect hyperalgesia (Fig. 3A, B). IL-10 did not have any significant effect on responses of the naive rat (Fig. 3).

The injection of IL-10 (1.0 ng) into the superficial caudal Vc significantly attenuated hyperalgesia on the ipsilateral side 30-60 min after IL-10 injection (p < 0.05) (Fig. 3C) when compared to saline control. However, this dose of IL-10 did not attenuate hyperalgesia on the contralateral side (Fig. 3D).

**Figure 4:**
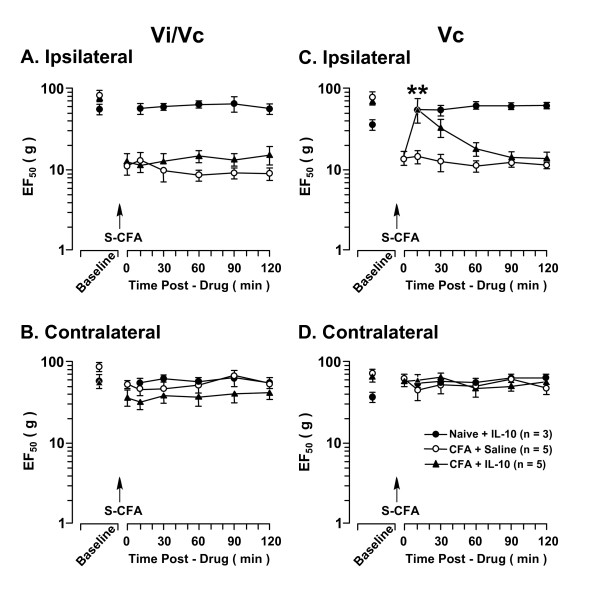
**The effect of IL-10 on orofacial hyperalgesia associated with cutaneous inflammation**. CFA was injected into the skin site overlying the right masseter muscle (S-CFA). At 24 h after CFA (time 0), there were significant decreases in the EF_50 _values ipsilateral to CFA injection (A, C), confirming the development of inflammatory hyperalgesia. There was no hyperalgesia developed at the contralateral site (B, D). IL-10 (1.0 ng) was injected into the Vi/Vc (A, B) or caudal Vc (C, D) in a volume of 500 nl after establishing behavioral hyperalgesia at 24 h after CFA. The attenuation of hyperalgesia was observed as a significant increase in EF_50_s after injection of IL-10. Injection of IL-10 into the Vi/Vc transition zone (A) did not attenuate cutaneous hyperalgesia. Injection of IL-10 into the caudal Vc (C) attenuated hyperalgesia, compared to saline (vehicle)-injected rats. Injection of IL-10 into either Vi/Vc or Vc did not produce an effect in non-inflamed (Naive) rats. Asterisks denote significant differences between the post-CFA (time 0) and after 1.0 ng IL-10 microinjection (p < 0.01). (ANOVA with repeated-measures and post hoc tests).

To compare the effect of IL-10 on masseter hyperalgesia versus cutaneous hyperalgesia, we injected CFA unilaterally into the skin side overlying the masseter to produce cutaneous inflammation and hyperalgesia. Cutaneous inflammation was only associated with hyperalgesia on the ipsilateral side and was assessed 1 d after CFA (Fig. 4). Injection of IL-10 (1.0 ng) into the ventral Vi/Vc did not attenuate cutaneous hyperalgesia (Fig. 4A). However, injection of the same dose of IL-10 into the caudal superficial Vc significantly increased EF_50_s, or reversed hyperalgesia, at 10 min after IL-10 (p < 0.01, n = 5) (Fig. 4C).

#### Effect of fluorocitrate

**Figure 5:**
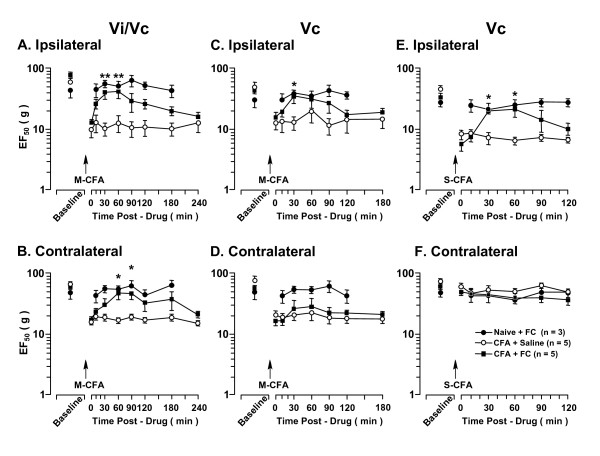
**The effect of fluorocitrate on CFA-induced orofacial hyperalgesia**. CFA was injected into the right masseter muscle (Masseter-CFA, A-D) or into the right cutaneous site overlying masseter muscle (S-CFA, E, F). Masseter inflammation was associated with bilateral hyperalgesia and only ipsilateral hyperalgesia was seen after cutaneous inflammation, tested at 24 h after inflammation immediately before the injection of the drug. Fluorocitrate (FC, 1.0 μg) was injected into the Vi/Vc (A, B) or caudal Vc (C-F) in a volume of 500 nl after establishing behavioral hyperalgesia at 24 h after CFA. The attenuation of hyperalgesia was observed as a significant increase in EF_50_s. Injection of fluorocitrate into the Vi/Vc transition zone attenuated hyperalgesia bilaterally after masseter CFA (A, B). Injection of fluorocitrate into the caudal Vc only attenuated masseter hyperalgesia on the ipsilateral site (C) and without an effect on contralateral hyperalgesia (D), compared to saline (vehicle)-injected rats. Injection of fluorocitrate into the caudal Vc attenuated cutaneous hyperalgesia (E) and without an effect on the contralateral side (F). Injection of fluorocitrate into either Vi/Vc or Vc did not produce an effect in non-inflamed (Naive) rats. Asterisks denote significant differences between the post-CFA (time 0) and after 1.0 ng IL-10 microinjection (*, p < 0.05; **, p < 0.01). (ANOVA with repeated-measures and *post hoc *tests).

We have shown recently that injection of a gliotoxin, fluorocitrate, into the ventral Vi/Vc transition zone produced dose-dependent attenuation of masseter hyperalgesia and inhibition of NMDA receptor phosphorylation after inflammation [17]. In the present study, at 1 d after injecting CFA into the *masseter *muscle, the attenuation of hyperalgesia was produced by focal microinjection of fluorocitrate (1.0 μg) into the ventral Vi/Vc transition zone or in the Vc via a chronically implanted guide cannula. Compared with post-CFA, the reductions in EF_50_s were reversed on the ipsilateral site starting at 30 min and lasting for 60 min after the fluorocitrate injection (1.0 μg; n = 5) into the ipsilateral Vi/Vc (Fig. 5A). The reversal of reduction in EF_50_s on the contralateral site was significant 60-90 min after fluorocitrate microinjection (1.0 μg; n = 5) into the ipsilateral Vi/Vc (Fig. 5B). On the other hand, the reduction in EF_50_s on the ipsilateral site, but not contralateral site, was significantly reversed at 30 min after fluorocitrate microinjection (1.0 μg; p < 0.05, n = 5) into the ipsilateral Vc, compared to post-CFA (Fig. 5C, D). At 1 d after injecting CFA into the *cutaneous *site, hyperalgesia developed ipsilaterally, but not contralaterally (Fig. 5E, F). The attenuation of cutaneous hyperalgesia was observed 30-60 min after injection of fluorocitrate (1.0 μg, p < 0.05, n = 5) into the Vc, whereas intra-Vi/Vc fluorocitrate did not produce an effect (1.0 μg, n = 5) (data not shown).

#### Effect of minocycline

**Figure 6:**
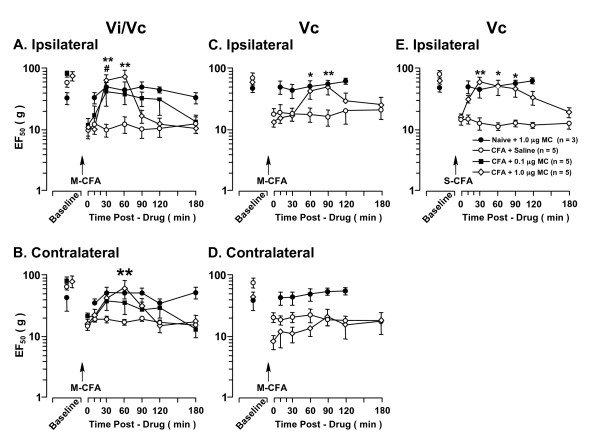
**The effect of minocycline on CFA-induced orofacial hyperalgesia**. CFA was injected into the right masseter muscle (Masseter-CFA, A-D) or into the right cutaneous site overlying masseter muscle (S-CFA, E). Masseter inflammation was associated with bilateral hyperalgesia and only ipsilateral hyperalgesia was seen after cutaneous inflammation, tested at 24 h after inflammation immediately before the injection of the drug. Minocycline (MC) was injected into the Vi/Vc (A, B) or caudal Vc (C-E) in a volume of 500 nl after establishing behavioral hyperalgesia at 24 h after CFA. The attenuation of hyperalgesia was observed as a significant increase in EF_50_s. Injection of minocycline into the Vi/Vc transition zone attenuated hyperalgesia bilaterally after masseter CFA (A, B). Injection of fluorocitrate into the caudal Vc only attenuated masseter hyperalgesia on the ipsilateral site (C) and without an effect on contralateral hyperalgesia (D). Injection of minocycline into the caudal Vc attenuated cutaneous hyperalgesia (E). Injection of minocycline into either Vi/Vc or Vc did not produce an effect in non-inflamed (Naive) rats. Asterisks denote significant differences between the post-CFA (time 0) and after 1.0 μg minocycline microinjection (*, p < 0.05; **, p < 0.01). Pound sign denotes significant difference between the post-CFA and after 0.1 μg minocycline microinjection (p < 0.05). (ANOVA with repeated-measures and *post hoc *tests).

Minocycline, a semisynthetic tetracycline, is an inhibitor of microglial activation and apparently has no direct action on astrocytes or neurons [47,48]. At 1 d after injecting CFA into the *masseter *muscle, the attenuation of hyperalgesia was observed after focal microinjection of minocycline (0.1 and 1.0 μg, n = 6/dose) into the ventral Vi/Vc transition zone. Compared with post-CFA values, the reduction in EF_50_s on the ipsilateral site was significantly reversed at 30 min (0.1 μg, p < 0.05 and 1.0 μg, p < 0.01) and 60 min (1.0 μg, p < 0.01) after minocycline injection (0.1 and 1.0 μg) into the ipsilateral Vi/Vc (Fig. 6A). The reduction in EF_50_s on the contralateral site was significantly reversed at 60 min after minocycline injection (1.0 μg, p < 0.01) into the Vi/Vc (Fig. 6B). On the other hand, after injection of minocycline (1.0 μg, n = 6) into the ipsilateral Vc, the reduction in EF50s on the ipsilateral, but not contralateral site, was significantly reversed at 60-90 min (Fig. 6C, D). At 1 d after injection of CFA into the *cutaneous *site, the attenuation of hyperalgesia was observed after injection of minocycline (1.0 μg) into the ipsilateral Vc (Fig. 6E), while intra-Vi/Vc minocycline did not produce an effect (1.0 μg; n = 6, data not shown).

Taken together (Table 1), microinjection of the antiinflammatory cytokine, IL-10, or glial inhibitors, fluorocitrate or minocycline, into the Vi/Vc transition zone attenuated orofacial hyperalgesia bilaterally after masseter inflammation. On the other hand, injection of these drugs into the superficial caudal Vc produced only ipsilateral attenuation of hyperalgesia after masseter inflammation. In contrast, cutaneous inflammation-induced orofacial hyperalgesia was attenuated by injecting IL-10, fluorocitrate and minocycline into the Vc but not the Vi/Vc site. Finally, these drugs had no effect on baseline responses of the non-inflamed (naive) rats.

**Table 1:** Summary of the effects of microinjection of IL-10, fluorocitrate and minocycline into the Vi/Vc or caudal Vc zone on CFA-induced masseter or cutaneous hyperalgesia.

	IL-10		Fluorocitrate		Minocycline		Saline	
	**Vi/Vc**	**Vc**	**Vi/Vc**	**Vc**	**Vi/Vc**	**Vc**	**Vi/Vc**	**Vc**

**Masseter-CFA**								

Ipsilateral	+	+	+	+	+	+	-	-

Contralateral	+	-	+	-	+	-	-	-

**Skin-CFA**								

Ipsilateral	-	+	-	+	-	+	-	-

Contralateral	-	-	-	-	-	-	-	-

**Naive**								

Ipsilateral	-	-	-	-	-	-	-	-

Contralateral	-	-	-	-	-	-	-	-

Plus signs (+) indicate significant attenuation of hyperalgesia. Minus signs (-) indicate a lack of effect on hyperalgesia. Note that intra-Vi/Vc injection of the three agents attenuated bilateral hyperalgesia associated with masseter inflammation without an effect on cutaneous hyperalgesia. Intra-Vc injection of the three agents only attenuated ipsilateral hyperalgesia after both masseter and cutaneous inflammation.

### The effects of microinjection of IL-10, fluorocitrate and minocycline on IL-1β mRNA transcription

We have shown previously that in response to masseter inflammation, there was a time-dependent upregulation of IL-1β, a prototype proinflammatory cytokine, in the trigeminal transition zone [17]. We next examined whether the effect of IL-10 and glial inhibitors on behavioral hyperalgesia was associated with an inhibition on IL-1β expression, a chemical mediator of inflammatory hyperalgesia in the Vi/Vc transition zone. Since the *in vivo *elimination half-life of IL-1β in rats is about 40 min [49], it is unlikely that a down-regulation of the existing IL-1β at the protein level would be detectable at the time when antihyperaglesic effect of the inhibitors occurred at 30-60 min after administration. Thus, we studied IL-1β mRNA transcription by RT-PCR.

**Figure 7:**
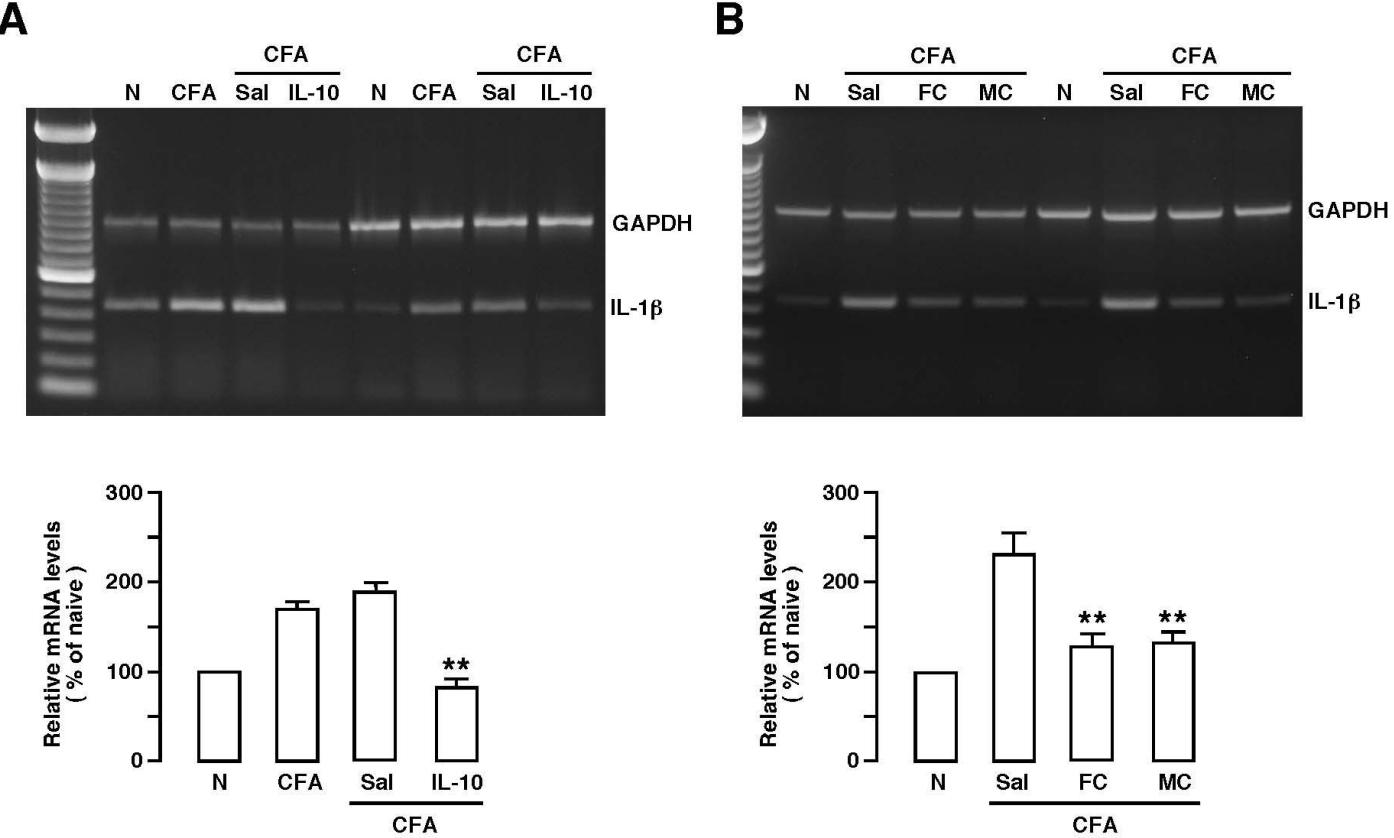
**Agarose gel electrophoresis (upper panel, two experiments) and quantitative comparison (lower panel) of RT-PCR-amplified products of the IL-1β mRNAs from rat Vi/Vc tissues**. A. Effect of IL-10. B. Effect of fluorocitrate (FC) and minocycline (MC). Compared to naive controls (N), there were significant increases in IL-1β mRNAs in the Vi/Vc transition zone after CFA-induced masseter inflammation, which was reversed in rats receiving IL-10, FC or MC. Asterisks denote significant differences from CFA or Sal-CFA, **, P < 0.01. Error bars represent S.E.M.

Following the same surgery and inflammation procedure as in the above behavioral experiment, Vi/Vc tissues were taken out at 30-60 min after microinjection of IL-10 (1.0 ng, n = 6), fluorocitrate (1.0 μg, n = 6) or minocycline (1.0 μg, n = 6) into the Vi/Vc transition zone. The primers used in this experiment targeted a 448-b segment of IL-1β mRNA (Table 2). Relative mRNA levels were compared after RT-PCR reactions. Masseter muscle inflammation induced a significant upregulation of IL-1β mRNA at 24 h after injection of CFA (p < 0.01) (Fig. 7). Compared to vehicle saline-injected rats (A, n = 6; B, n = 7), the IL-1β levels were reduced to the control level after injection of IL-10 (Fig. 7A), fluorocitrate or minocycline (Fig. 7B) into Vi/Vc.

**Table 2:** Polymerase chain reaction primers

Gene	Accession No./Positions	Sense/Antisense	Sequences	Product (bp)
GAPDH	X02231/76-101	Sense	TGA AGG TCG GTG TGA ACG GAT TTG GC	983
	X02231/1058-1035	Antisense	CAT GTA GGC CAT GAG GTC CAC CAC	

IL-1β	NM_031512/345-364	Sense	GCA CCT TCT TTT CCT TCA TC	448
	NM_031512/792-773	Antisense	CTG ATG TAC CAG TTG GGG AA	

### Effects of masseter and skin inflammation on NMDA receptor phosphorylation and GFAP protein levels

**Figure 8:**
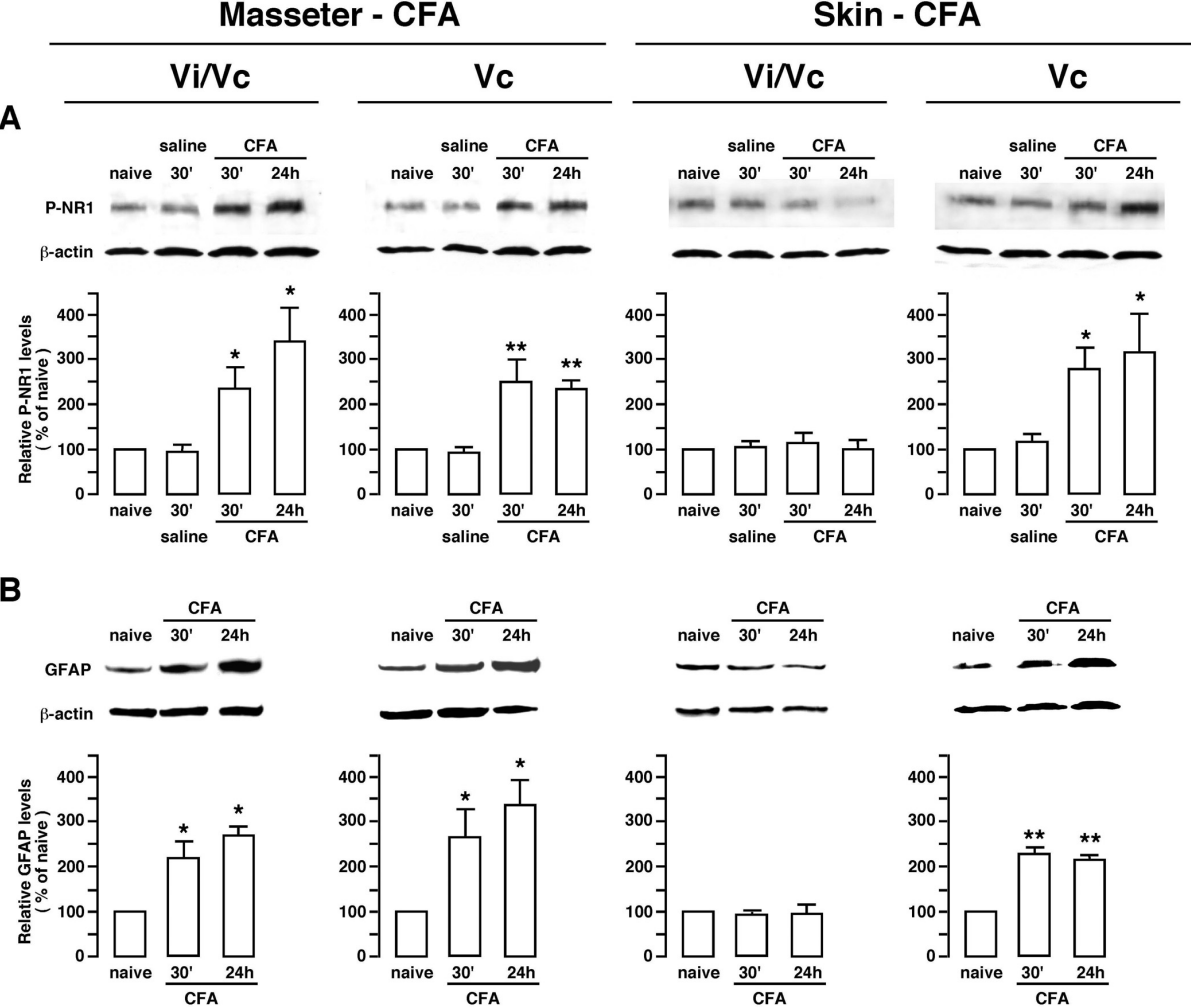
**Western blots illustrating enhanced NMDA receptor phosphorylation and upregulation of GFAP levels in the trigeminal Vi/Vc transition zone and caudal Vc after masseter or skin inflammation**. The examples of immunoreactive bands for phosphoserine896 NR1 subunit (P-NR1) of the NMDA receptor (A) and GFAP (B) are shown on top of each panel. CFA was injected into the masseter (Masseter-CFA) or the overlying skin site (Skin-CFA) to produce deep or cutaneous inflammation, respectively. Note that compared to naive and saline controls, masseter inflammation induced an increase in P-NR1 and GFAP levels in both Vi/Vc and caudal Vc at 30 min (30') and 24 h after CFA (Left). However, cutaneous inflammation only led to an increase in P-NR1 and GFAP levels in caudal Vc, but not in Vi/Vc transition zone (Right). *, p < 0.05; **, p < 0.01 vs. naive rats. N = 3-4 for each time point. Error bars represent S.E.M.

To verify differential involvement of the trigeminal Vi/Vc transition zone and caudal laminated Vc in response to deep or cutaneous orofacial tissue injury, we examined NMDA receptor phosphorylation and upregulation of GFAP, a marker of reactive astrocytes after masseter and cutaneous inflammation. NMDA receptors are prominently involved in persistent pain and exhibit enhanced subunit phosphorylation after injury [50-52]. We have shown that masseter inflammation induced a significant increase in NMDA receptor NR1 subunit phosphorylation and GFAP levels in the Vi/Vc transition zone [17]. Injection of the NMDA receptor antagonist into the Vi/Vc transition zone attenuated orofacial hyperalgesia after masseter inflammation [7]. Here we compared the effects of masseter inflammation with that of inflammation of the skin site overlying the masseter muscle. As shown in Fig. 8, injection of CFA into the masseter produced significant increases in NR1 serine 896 phosphorylation (Fig. 8A left) and GFAP levels (Fig. 8B left) in both Vi/Vc and caudal Vc. In contrast, injection of CFA into the cutaneous site only produced an increase in NMDA receptor phosphorylation (Fig. 8A right) and GFAP (Fig. 8B right) levels in the Vc without an effect in Vi/Vc.

## Discussion

The aims of the present study were two-fold. First, we examined the effects of antiinflammatory cytokine and glial inhibitors on orofacial hyperalgesia following focal microinjection into the subregions of the spinal trigeminal complex. Second, we further established the role of the trigeminal Vi/Vc transition zone in the development of hyperalgesia associated with deep tissue injury. After systematic comparison, it is clear that injection of an antiinflammatory cytokine, IL-10, and two glial inhibitors, fluorocitrate and minocycline, into the Vi/Vc transition zone attenuated hyperalgesia after masseter muscle inflammation, but had no effect on hyperalgesia after cutaneous inflammation. On the other hand, injection of these agents into the caudal Vc attenuated hyperalgesia associated with both masseter and cutaneous inflammation. Western blot data showed that masseter inflammation induced an enhanced NMDA receptor phosphorylation and upregulation of GFAP in both Vi/Vc and Vc zones, while cutaneous inflammation only induced similar effects in the caudal Vc. These findings support the view that glial activation and cytokine activity are important factors in persistent pain development and the Vi/Vc transition zone provides additional processing for deep orofacial input.

Cytokines are a complex group of proteins able to exert pleiotropic effects on activity of a variety of cells. Together with a group of so-called *proinflammatory cytokines *including tumor necrosis factor (TNF), IL-1, IL-6 and IL-8, a variety of *antiinflammatory cytokines *including IL-10 and the IL-1 receptor antagonist (IL-1ra) are also produced during humoral and cell-mediated immune responses [53]. Interleukin-10 is induced in the CNS in a number of disease conditions [54]. Analysis of cytokine mRNA expression in the CNS of mice with experimental autoimmune encephalomyelitis reveals that IL-10 mRNA expression correlates with recovery [55].

Interleukin-10 acts on immune cells to suppress the release of IL-1 and TNF-α [56]. Interleukin-1ra, an analogue of IL-1, antagonizes IL-1 activity by competing for IL-1 receptors. We have previously shown that IL-1ra attenuated inflammation-induced NMDA receptor phosphorylation and hyperalgesia [17,57]. The present study further found that IL-10 produced antihyperalgesia and inhibition of IL-1β transcription in a rat model of orofacial inflammatory pain when injected into the identified brain stem sites involved in trigeminal pain processing. These results are consistent with the antinociceptive and neuroprotective effects of IL-10 at the spinal level and support the use of IL-10 as a therapeutic agent against persistent pain [34,35,58-60].

The use of two glial inhibitors in the present study produced attenuation of orofacial hyperalgesia. These observations are consistent with the literature [61-63]. Minocycline selectively inhibits microglia [47,48]. Fluorocitrate has been shown to be relatively selective against astroglia [19,64]. Our results support the current view that central glial activation plays a role in orofacial hyperalgesia [17,19,20] or persistent pain in general [22]. It appears that the involvement of glia occurred at multiple levels since the injection of glial inhibitors into the two subregions of the spinal trigeminal complex was effective in attenuating hyperalgesia. Evidence suggests that glia are intimately involved in the control of neuronal activity [65,67]. Glial TNF-α modulates synaptic strength in the brain [68,69]. However, the functional consequence of glial activation is not fully understood. Models of peripheral nerve injury and neuropathic pain have been associated with a decrease in astrocytic glutamate transporter activity [70-73] and astroglia are apparently activated in response to peripheral nerve or tissue injury [17,74]. Thus, there is a reciprocal relationship between the astrocytic activation state after nerve injury and astrocytic glutamate transporter GLT-1 expression. In primary astrocytic cultures that exhibit an activated phenotype, propentofylline, a glial modulator, induces glutamate transporter GLT-1 expression and glutamate uptake [75]. Since astroglial glutamate transporters play an important role in maintaining an appropriate level of glutamate extracellular concentration, a reduction in GLT-1 glutamate transporter expression may lead to a build up of glutamate concentration in the synaptic cleft, resulting in neuronal hyperexcitability and behavioral hyperalgesia.

After masseter inflammation, IL-1β is selectively induced in astroglia in the Vi/Vc region [17], although previous studies have indicated that IL-1β is produced primarily in microglia [76]. It is interesting that both glial inhibitors suppressed inflammation-induced IL-1β mRNA upregulation. These results suggest interaction between microglia and astroglia in the mechanisms of inflammatory hyperalgesia. Studies have suggested that microglial activation precedes activation of astrocytes [see [18], for a review]. Activation of TLR4 on microglial cells may lead to astroglial activation [77]. Kawasaki et al. [78] showed that early microglial and later astroglial activation were associated with neuropathic pain. Recent studies suggest that IL-18, a member of the IL-1 cytokine family, is released from microglia and acts on the IL-18 receptor on astrocytes [79]. The IL-18-mediated microglia-astroglia interaction potentiates neuropathic pain behavior in rats; and inhibition of IL-18 signaling pathways suppresses astroglial activity and nerve injury-induced allodynia [79]. Taken together, these findings support the view that coordinated activation of microglia and astroglia contribute to the development and maintenance of persistent pain.

The present study showed that orofacial inflammatory hyperalgesia was attenuated by injection of an antiinflammatory cytokine and two glial inhibitors into the spinal trigeminal complex, associated with a reduction of IL-1β mRNAs. However, these drugs did not produce an effect on baseline responses before inflammation, suggesting that cytokine and glial activity do not contribute to pain processing under normal conditions. While these data support the involvement of both glia and inflammatory cytokines in persistent pain, the mechanisms of interactions between IL-10 and glia during the pain processing are only speculative. Interleukin-10 not only inhibits synthesis of proinflammatory cytokines by macrophages/microglia, it also induces anergy in brain-infiltrating T cells [54]. The activation of astroglia may be suppressed by IL-10 indirectly through inhibition of cytokine production [80]. We have shown recently that astroglial activation and IL-1β upregulation in the Vi/Vc transition zone contributes to trigeminal responses to deep orofacial tissue inflammation and hyperalgesia [17]. The inhibition of IL-1β mRNA transcription by IL-10 and glial inhibitors would be consistent with their antihyperalgesic effect. Importantly, IL-10 suppresses p38 mitogen-activated protein kinase activation in immune cells [81], which may be a key interface between IL-10 and glial cells in producing antihyperalgesia [see [56]].

Through a systematic comparison of the effects of IL-10, fluorocitrate and minocycline on masseter and cutaneous hyperalgesia, we further demonstrate a selective involvement of the trigeminal Vi/Vc transition zone in response to deep orofacial tissue injury. Injection of these agents into the Vi/Vc transition zone only attenuated hyperalgesia after masseter inflammation without an effect on cutaneous hyperalgesia. In contrast, both masseter and cutaneous hyperalgesia were attenuated after injection of these agents into the caudal Vc, which is consistent with a role of laminated Vc in trigeminal pain processing. These results are in good agreement with our previous study where an NMDA receptor antagonist was injected [7]. These observations are further supported by Western blot analysis that shows a selective enhancement of NMDA receptor phosphorylation and GFAP upregulation in the Vi/Vc transition zone after masseter inflammation. The findings strengthen the view that while both deep and cutaneous orofacial nociceptive input are processed in the laminated Vc, the trigeminal Vi/Vc transition zone is also involved in integrating responses to deep tissue injury [82].

One important feature of neuronal activation in the Vi/Vc transition zone is that a unilateral injury always produces bilateral activation, particularly in the ventral Vi/Vc [8,10,1,13]. This is in sharp contrast to caudal laminated Vc where a predominantly unilateral neuronal activation is always observed [11]. Interestingly, contralateral as well as ipsilateral hyperalgesia developed after a unilateral injection of CFA into the masseter. In contrast, unilateral cutaneous inflammation only produced hyperalgesia on the ipsilateral side. It appears that contralateral Vi/Vc activation underlies the development of contralateral hyperalgesia, which should be categorized as secondary hyperalgesia or pain referred to a site remote from the injury. We also observed for the first time that unilateral administration of IL-10, fluorocitrate or minocycline into the Vi/Vc transition zone was able to attenuate ipsilateral, as well as contralateral, hyperalgesia after masseter inflammation. These results suggest an indirect or polysynaptic circuitry from the ipsilateral to the opposite Vi/Vc. An important relay is likely in the rostral ventromedial medulla (RVM), the pivotal structure in descending pain modulation including inhibition and facilitation [83]. Sugiyo et al. [14] have shown a reciprocal pain facilitatory circuitry between the RVM and Vi/Vc transition zone. Lesions of the RVM eliminate masseter hyperalgesia on both sides [14]. Neuronal activation in the Vi/Vc is also modulated by input from the caudal Vc [14,84-86]. However, injection of either one of the three drugs into the Vc only reduced ipsilateral hyperalgesia without an effect on contralateral hyperalgesia. This would suggest that, with regard to secondary hyperalgesia, facilitation from the RVM-Vi/Vc circuitry is sufficient and necessary.

## Conclusion

These findings support the view that glial activation and cytokine activity are important factors in persistent pain development and the Vi/Vc transition zone provides additional processing for deep orofacial input. The glial inhibition and interruption of the cytokine cascade after inflammation may provide pain relief.

## List of Abbreviations

Bupi: bupivacaine; CFA: complete Freund's adjuvant; CNS: central nervous system; EF_50_: the effective force that produces 50% response frequency; FC: fluorocitrate; GFAP: glial fibrillary acidic protein; IL: interleukin; IL-1ra: IL-1 receptor antagonist; Mass: masseter muscle; MC: minocycline; NMDA: N-methyl-D-aspartate; RVM: rostral ventromedial medulla; Sal: saline; S-R: stimulus-response; Vc: subnucleus caudalis; TNF: tumor necrosis factor; Vi: subnucleus interpolaris

## Competing interests

The authors declare that they have no competing interests.

## Authors' contributions

KS is involved in the experimental design, carried out the behavioral, pharmacological and Western blot studies, and drafted the manuscript. WG is involved in the experimental design and Western blot studies. HW is involved in the design and biochemical experiments including Western blot and RT-PCR. SPZ is involved in behavioral testing and RT-PCR experiment. SCL contributed to the design and behavioral pharmacology experiments. KI is involved in experimental design and drafting the manuscript. FW contributed to the design and behavioral pharmacology experiments and participated in drafting the manuscript. RD and KR conceived of the study, and participated in its design and coordination and helped to draft the manuscript. All authors read and approved the final manuscript.
